# SCIENCE AND CARE PARTNERSHIPS: THE ROLE OF THE SCIENTIFIC LINKING PIN RESEARCHER

**DOI:** 10.1093/geroni/igad104.2857

**Published:** 2023-12-21

**Authors:** Reena Devi, Irma Everink, Judith Urlings, Alys Griffiths, Hilde Verbeek, Kirsty Haunch, Karen Spilsbury, Jan Hamers

**Affiliations:** University of Leeds, Leeds, England, United Kingdom; Maastricht University, Maastricht, Limburg, Netherlands; Living Lab in Ageing and Long-Term Care, Maastricht, Limburg, Netherlands; University of Sheffield, Sheffield, England, United Kingdom; Maastricht University, Maastricht, Limburg, Netherlands; University of Leeds, Leeds, England, United Kingdom; University of Leeds, Leeds, England, United Kingdom; CAPHRI, Maastricht University, Maastricht, Limburg, Netherlands

## Abstract

**Background:**

Science and care partnerships work on promoting quality of life, care and work in long-term-care (LTC) settings. The Scientific Linking Pin (SLP) is an academic employed by a university and works one day per week in a LTC organisation. The SLP role is pivotal to the success of partnerships; the nature of this role has not yet been captured.

**Methods:**

Fifteen SLPs took part in semi-structured interviews. Data were thematically analysed.

**Results:**

Data are presented over two themes. The first theme, the SLP experience, outlines that SLPs have rich insights into what matters to, and the challenges experienced in LTC organisations. SLPs are well positioned to impact practice, and develop their professional careers. SLPs do experience doubts and insecurities and require mentorship/supervision. The second theme, navigating in a SLP role, outlines the multifaceted tasks involved: relationship building, raising awareness, identifying priorities, generating research questions, building committees, brokering knowledge, developing research studies, generating academic output, building links and connections, and assisting with internal projects. The challenges SLPs face in their work include mistrust and poor engagement from care staff, working with staff from different professional backgrounds, research not being a priority, multiple and rapidly changing priorities, and differences in expectations. Relationship building, creating a ‘safe’ space for care staff, building engagement, and expectation management helped to address these challenges.

**Conclusion:**

Partnership working in the care sector is gaining international recognition and adoption. This study described one key role that contributes to the success of the partnership model: the SLP.

